# Positive Illusions: The Role of Cognitive Distortions Related to Gambling and Temporal Perspective in Chasing Behavior

**DOI:** 10.1007/s10899-021-10068-5

**Published:** 2021-08-20

**Authors:** Giovanna Nigro, Olimpia Matarazzo, Maria Ciccarelli, Barbara Pizzini, Mariagiulia Sacco, Marina Cosenza

**Affiliations:** Department of Psychology, Università degli studi della Campania “Luigi Vanvitelli”, Viale Ellittico 31, 81100 Caserta, Italy

**Keywords:** Gambling, Gambling disorder, Chasing behavior, Gambling-related cognitive distortions, Temporal perspective

## Abstract

Chasing, or continuing to gamble to recoup previous losses, is a behavioral marker and a diagnostic criterion for gambling disorder. Even though chasing has been recognized to play a central role in gambling disorder, research on chasing is still relatively scarce. This study first empirically investigated the interplay between cognitive distortions related to gambling, temporal perspective, and chasing behavior in a sample of habitual gamblers. Two hundred and fifty-five adults took part in the study. Participants completed the South Oaks Gambling Screen (SOGS), the Gambling Related Cognitions Scale (GRCS), the 14-item Consideration of Future Consequences scale (CFC-14), and performed a computerized task assessing chasing behavior. Participants were randomly assigned to three experimental conditions (Control, Loss, and Win). Hierarchical logistic regression analysis showed that the decision to chase depended on scores on the CFC-14 Immediate scale and the GRCS dimensions Gambling Expectancies and Interpretative Bias. Hierarchical linear regression analysis indicated that, chasing frequency was affected by Loss condition, distortions related to gambling expectancies and predictive control, as well as by myopia for the future. Interestingly, the results of path analysis clearly indicated that some cognitions related to gambling predict chasing frequency not only directly, but also indirectly via shortened time horizon. Notably, gambling severity did not predict either the decision to chase, or the chasing persistence. These findings provide further evidence that nonchasers and chasers seem to belong to two quite distinct subtypes of gamblers. Such a difference could be useful for targeting more effective intervention strategies in gambling disorder treatment.

## Introduction

Cognitive distortions (or cognitive biases) refer to irrational ways of thinking that foster problematic behaviors (Fortune & Goodie, 2012; Goodie and Fortune, 2013; Goodie et al., 2019). Gambling-related cognitions are distortions specific to gambling behavior. According to Raylu and Oei (2004), they can be conceptualized as erroneous cognitions about success at gambling as well as beliefs about self in relation to gambling that motivate people to gamble and continue gambling, despite persistent losses. A large body of research has demonstrated that gambling-related cognitions represent a significant predictor of gambling behavior among both adults and adolescents (Ciccarelli et al., 2016, 2017; Ciccarelli et al., 2020; Clark et al., 2014; Cosenza et al., 2019a, 2019b; Cosenza & Nigro, 2015; Fortune & Goodie, 2012; Ledgerwood et al., 2020; Matarazzo et al., 2019; Mathieu et al., 2018; for a recent review, see Labrador et al., 2020).

Since gambling-related cognitions are biases that reframe gambling outcomes in such a way as to encourage the continuation of gambling, “cognitive distortions result in individuals overestimating personal skills and probabilities of winning and lead to further attempts to recoup losses through continued gambling” (Hunt and Blaszczynski, 2019, p. 18). Consequently, they could play a crucial role in chasing behavior, that is continuing gambling to recoup previous losses (Lesieur, 1979). Note that since the publication of the third edition of the Statistical Manual of Mental Disorders (DSM-III-R; American Psychiatric Association [APA], 1987), chasing losses has been considered a behavioral marker and a defining feature of disordered gambling and a hallmark of the transition from recreational to disordered gambling (Zhang & Clark, 2020).

Following the DSM, chasing implies returning to gamble on another day in the hope of recouping lost money. However, chasing is not confined, as DSM criteria would seem to suggest, to between-session chasing (i.e., returning on a later day to recoup lost money). Chasing also refers to the tendency to gamble too long within a gambling session (within-session chasing; Breen & Zuckerman, 1999, p. 1080). Even if originally chasing refers mainly to continue gambling to recoup previous losses starting a new gambling session, subsequent research also focused on chasing wins, that is, to continue gambling after a win in the hope to gain more (e.g., Blaszczynski & Nower, 2002; O’Connor & Dickerson, 2003; Subramaniam et al., 2017). For instance, Blaszczynski and Nower’s pathways model (2002) assumes that there are two forms of chasing, namely chasing losses and chasing wins, since chasing behavior refers to persistent gambling both when losing or winning within a gambling session (Goodie et al., 2019).

Prior studies have indicated that chasing is associated with impulsivity (Breen & Zuckerman, 1999), sensation seeking (Linnet et al., 2006), an increased activation in brain regions related to reward expectation (Campbell-Meiklejohn et al., 2008), low sensitivity to punishment (Kim & Lee, 2011), poor decision-making (Nigro et al., 2018a), disinhibition (Nigro et al., 2018b), alexithymia (Bibby, 2016), deficit in mentalization (Cosenza et al., 2019a, 2020), and heightened levels of craving (Ciccarelli et al., 2019b; Cosenza et al., 2020). Interestingly, Campbell-Meiklejohn et al. (2008) found an association between chasing behavior, high scores on the *Interpretative Bias* dimension of the Gambling Related Cognitions Scale (GRCS; Raylu & Oei, 2004) and increased activity in cortical areas linked to incentive motivation and reward anticipation. Ciccarelli, et al. (2019a) have found a significant positive association between chasing and shortened time horizon, showing that chasers differ significantly from nonchasers in terms of temporal perspective, with chasers being more focused on the present rather than on the future consequences of their behavior. Notably, recent research has shown that chasers and nonchasers represent two distinct subgroups of gamblers, over and above gambling severity (Ciccarelli et al., 2019a, 2019b; Cosenza et al., 2020; Nigro et al., 2018a, 2018b, 2019; see also Linnet et al., 2006).

Although the increasing acknowledgment that people can continue gambling either in the hope of recoup previous losses or gaining more money, only three studies have compared chasing losses and chasing wins (Lister et al., 2016; Nigro et al., 2019; O’Connor & Dickerson, 2003). O’Connor and Dickerson (2003), who first analyzed the role of chasing in relation to impaired control over gambling, found no difference between returning later to chase after large wins or after losing. Lister et al. (2016) showed that gamblers with higher winning money motivation were more likely to decide to chase and chased more in response to both losses and wins. Unlike O’Connor and Dickerson (2003) and Lister et al. (2016), who did not observe significant differences between chasing losses and chasing wins, Nigro et al. (2019) reported that people chased more and more frequently to increase their winnings. Overall, the findings from chasing literature seem to corroborate the idea that “problem gamblers have difficulty quitting, regardless of whether they are losing or winning” (Breen & Zuckerman, 1999, p. 1098).

The aim of the present study was to investigate for the first time the interplay between chasing behavior, cognitive distortions related to gambling, and temporal perspective in a sample of adult habitual gamblers. As chasing behavior was found associated with some cognitive biases (Campbell-Meiklejohn et al., 2008), we expected that high levels of gambling cognitions would be associated with chasing proneness. Consistent with the results of a recent research showing a correlation between chasing and weak concern for the long-term consequences of engaging in the behavior (Ciccarelli et al., 2019a), it is also hypothesized that chasers will demonstrate a weaker future time orientation compared to nonchasers. Consistent with recent findings (Nigro et al., 2019) we would expect that chasing behavior vary as a function of experimental condition. A further purpose of the study was to clarify through path analysis if the impact of present orientation on chasing was mediated by cognitive biases or if present orientation was on the path from cognitive distortions to chasing.

## Materials and Methods

### Participants

Two hundred and fifty-five adults (69.8% males) aged between 18 and 82 years (Mage = 31.23 years; SD = 14.03) participated in the study. Data were collected from September 2019 to February 2020, that is before Italy’s Covid-19 lockdown. The sample was recruited from several Video Lottery terminal venues in Southern Italy, offering the same wide range of gambling activities. The percentage of people contacted who refused to participate in the study was about 32%. The two inclusion criteria were as follows: (1) participants reported to gamble once a week or more, and (2) were 18 years of age or over. Of the volunteers who accepted to participate in the experimental study 64.8% were single, 22.2% married, 10.9% separated or divorced, and 2.1% widowed. About modal occupation status, 29.7% of the participants were unemployed, 20.1% manual workers, and 18.6% office workers.

Participants were tested on-site, in a quiet room made available by the management and did not receive anything for participating in the study. As the study included an experimental chasing task with three conditions (Control, Loss, and Win), an equal number of participants (*N* = 85) was randomly assigned to each condition. Such a task was administered via computer. Participants also completed three paper-and pencil measures, for each of which they received detailed written instructions. Half of the participants completed the chasing task at the beginning of the session, the other half at the end. In such a way, the (potential) influence of the experimental task on the paper-and-pencil measures, and vice versa, was balanced.

### Measures

Participants were administered the South Oaks Gambling Screen (SOGS; Lesieur & Blume, 1987; Italian translation: Cosenza et al., 2014), the Gambling Related Cognitions Scale (GRCS; Raylu & Oei, 2004; Italian validation: Iliceto et al., 2015), the Consideration of Future Consequences Scale (CFC-14; Joireman et al., 2012; Italian validation: Nigro et al., 2016), and an experimental computerized task developed to assess chasing behavior (Nigro et al., 2018a).

The SOGS is a self-report measure of the frequency and the severity of gambling problems. The questionnaire is composed of 20 scored items and some unscored items. The total score varies from 0 to 20. Scores of 0–2 indicate no problem gambling, scores of 3–4 reflect problem gambling, and scores of 5 or above denote (probable) pathological gambling. The unscored items request participants to indicate, among others, the frequency of participation in different gambling activities (“not at all,” “less than once a week,” or “once a week or more”), and the largest amount of money gambled in 1 day. Furthermore, we asked participants to indicate the main reasons for gambling in a list of motives (Volberg, 1993). The SOGS was found to have a high internal consistency reliability coefficient in this study (Cronbach’s α = 0.81). Although the SOGS has been found to produce inflated pathological gambling estimates, it is still frequently used as a screen in experimental research (James et al., 2016). In this study, the SOGS was chosen to allow comparisons with previous studies on chasing behavior (e.g., Breen & Zuckerman, 1999; Campbell-Meiklejohn et al., 2008; Ciccarelli et al., 2019a, 2019b; Linnet et al., 2006; Nigro et al., 2018a, 2018b, 2019).

The GRCS is a 23-item questionnaire assessing the susceptibility to common gambling distortions and beliefs on five subscales. In addition to an overall GRCS score, the questionnaire’s five subscales evaluate specific aspects of gambling-related cognitive distortions. In particular, the *Gambling-related Expectancies* (GE) subscale focuses on expected benefits from gambling; the *Illusion of Control* (IC) dimension reflects cognitions relating to ability to control gambling outcomes; the *Predictive Control* (PC) factor focuses on probability errors (such as gambler's fallacy); the *Inability to Stop gambling* (IS) subscale refers to respondents' perceived inability to control their gambling behavior. Finally, the *Interpretative Bias* (IB) dimension reflects cognitions relating to reframing gambling outcomes to encourage further play. Participants are requested to indicate the extent to which they agree with each statement on a 7-point scale, with higher scores reflecting higher scores reflecting higher gambling-related expectancies and cognitive distortions. For the present study Cronbach’s alphas for the subscales were as follows: GE = 0.71; IC = 0.78; PC = 0.85; IS = 0.80; IB = 0.83. The Cronbach’s α for the full scale was 0.93.

The CFC-14 measures individual differences in the extent to which people weigh the immediate as opposed to distant implications of current behaviors and events. Responses are made with a 7-point Likert scale ranging from 1 (extremely uncharacteristic of me) to 7 (extremely characteristic of me). Items are equally divided into two subscales: Immediate (CFC-I) that concerns orientation toward the present, and Future (CFC-F) that concerns the consideration of the future consequences of actual behavior. The Cronbach's alphas for the Immediate and Future scales were 0.85 and 0.82, respectively, in a large sample of Italian adults (Nigro et al., 2016). For the present study, the Cronbach's alphas were 0.86 (Immediate Scale) and 0.80 (Future scale).

Chasing behavior was measured by means of a 60-trial computerized task (ChasIT; Nigro et al., 2018a) simulating a card game in which participants played against the house (about the validity of the behavioral task see Nigro et al., 2019). The initial amount of (virtual) money was 10 Euros and participants were asked to treat the initial stake as real money. Each trial presented two cards, each one reporting a number ranging from 1 to 9. Participants were told they would win €1, if they had the highest card, but lose €1, if the house had the highest card. For each of the first 30 trials (first phase), participants received a positive (“You won €1!”) or a negative (“You lost €1!”) feedback.

After the first phase, in the Control condition participants kept the entire budget, in the Win condition earned two euros more than the initial budget, whereas in the Loss condition lost €12 (i.e., two euros more than the initial budget). At the end of the first phase, participants were requested to decide if they would like to continue playing or to stop, regardless of experimental condition.

During the second phase (30 trials), after each trial, participants received the following feedback: “You won (or lost) 1 Euro! Now, your credit is X Euros. If you want to continue playing, please, press the key ‘M’ on the computer keyboard. If you decide to stop playing, please, press the key ‘Z’ on the computer keyboard.” In such a way, participants could decide, in any moment, if they would like to continue or stop the game, simply by pressing the designated key.

As a function of experimental condition, wins and losses were randomly distributed throughout the gambling sessions (15 and 15 in the Control condition, 9 and 21 in the Loss condition, 21 and 9 in the Win condition), but the sequence of wins and losses was the same for every participant in that condition. At the end of the second phase the final budget was €10 in the Control condition, minus €14 in the Loss condition, and €24 in the Win condition. Participants who decided to quit the game at the beginning of the second phase were considered nonchasers, whereas participants who chose to continue gaming were classified as chasers. Since participants could play till the end, the highest chasing total score was 30. Similar to Lister et al.’s procedure (2016), participants who chose not to chase were coded as playing for ‘0’ chasing spins.

The decision to quit or continue gaming at the end of the first phase (choice to chase) and the total number of trials played during the second phase (chasing frequency) were the two dependent measures of interest.

### Statistical Analyses

Data analyses were conducted using IBM SPSS version 20. The α level was set at *p* < 0.05. All variables were initially screened for missing data, distribution abnormalities, and outliers (Tabachnick & Fidell, 2019). Since the distributions of chasing frequency, SOGS and GRCS scores were positively skewed, square-root transformation was performed on these variables, so that assumptions of normality, linearity, and homoscedasticity had been adequately met. Using *p* < 0.001 criterion for Mahalanobis distance, three participants were eliminated as clear multivariate outliers. This left a final sample size of 255.

Pearson correlation coefficients were calculated to examine the relationships among the study variables. Analysis of variance was used to assess mean differences on continuous variables. For categorical data, differences in percentages were compared with the chi-square test. Logistic and linear regression analyses were performed to examine the unique contribution of predictor variables to chasing behavior. To control for the presence of multicollinearity, before interpreting the regression coefficients, we calculated the variance inflation factors (VIF).

Considering the results of the linear regression analysis and to clarify if the impact of present orientation on chasing was mediated by gambling-related cognitions or if present orientation was on the path from cognitive distortions to chasing, path analysis was conducted with the EQS 6.2 software program for structural equation modeling (Bentler, 2008). For each estimated model, goodness of model fit was evaluated with the likelihood ratio chi-square test statistic corrected for data nonnormality with Satorra and Bentler's (1994) method (S-B χ^2^), as well as with four descriptive fit indices: the standardized root-mean square residual (SRMR), the root-mean-square error of approximation (RMSEA) with its 90% confidence interval (90% CI), the goodness of fit index (GFI), and the comparative fit index (CFI). Acceptable fits between model and data are reflected by a non-significant S-B χ^2^, GFI and CFI indexes of 0.95 or greater, RMSEA of between 0.05 and 0.08.

## Results

Means and standard deviations by experimental condition and gender are presented in Table 1.

**Table 1:** Means and standard deviations by experimental condition and gender

Condition	Control (*N* = 85)				Loss (*N* = 85)				Win (*N* = 85)			
Gender	M (*N* = 59)		F (*N* = 26)		M (*N* = 63)		F (*N* = 22)		M (*N* = 56)		F (*N* = 29)	
	M	*SD*	M	*SD*	M	*SD*	M	*SD*	M	*SD*	M	*SD*
SOGS^a^ Total score*	1.50	0.60	1.12	0.32	1.41	0.61	1.21	0.47	1.67	0.68	1.12	0.32
GRCS^b^												
Gambling Expectancies (GE)*	2.46	0.56	2.16	0.36	2.50	0.65	2.19	0.33	2.64	0.63	2.22	0.45
Illusion of Control (IC)*	2.27	0.47	2.06	0.17	2.34	0.67	2.29	0.68	2.44	0.62	2.08	0.22
Predictive Control (PC)*	3.18	0.65	2.68	0.42	3.19	0.88	3.00	0.96	3.29	0.83	2.67	0.37
Inability to Stop gambling (IS)*	2.65	0.59	2.39	0.35	2.67	0.74	2.58	0.66	2.73	0.59	2.39	0.40
Interpretative Bias (IB)*	2.64	0.75	2.11	0.26	2.55	0.72	2.39	0.72	2.60	0.80	2.17	0.45
GRCS Total score*	5.99	1.17	5.13	0.61	6.02	1.42	5.63	1.46	6.20	1.40	5.20	0.75
CFC-14^c^												
Immediate	20.66	7.84	18.65	6.77	20.76	7.32	17.91	6.93	22.14	7.75	18.48	8.15
Future	31.49	8.06	32.08	9.29	32.49	7.77	33.00	7.22	32.93	8.62	31.07	9.87
Chasing frequency*	1.35	0.67	1.13	0.40	1.64	1.15	1.57	1.21	1.46	0.94	1.28	0.69

^a^South Oaks Gambling Screen

^b^Gambling Related Cognitions Scale

^c^Consideration of Future Consequences-14 Scale

^*^Transformed scores

Preliminarily, to ascertain whether participants assigned to the three experimental conditions differed in terms of gender, age, education, SOGS, GRCS, and CFC-14 scores, data were submitted to χ^2^ test or univariate ANOVA. The results indicated that the three groups did not differ each other regarding gender, age, and education, nor in terms of SOGS, GRCS, and CFC-14 scores (all *ps* ns). Percentages of participants who decided to chase were as follows: 28.6% in the Control condition, 37.1% in the Loss condition, and 34.3% in the Win condition, respectively.

To ascertain whether SOGS, GRCS, and CFC-14 scores varied by gender, data were submitted to univariate ANOVA. With the only exception of scores on the CFC-14 Future subscale, effects of gender were observed on the SOGS (*F*_1, 254_ = 23.9; *p* =  < 0.001; η_p_^2^ = 0.086), the GRCS dimensions (GE: *F*_1, 254_ = 19.79; *p* =  < 0.001; η_p_^22^ = 0.073; IC = : *F*_1, 254_ = 8.54; *p* =  < 0.01; η_p_^2^ = 0.033; PC:: *F*_1, 254_ = 19.69; *p* =  < 0.001; η_p_^2^ = 0.072; IS: *F*_1, 254_ = 8.66; *p* =  < 0.01; η_p_^2^ = 0.033; IB:: *F*_1, 254_ = 17.04; *p* =  < 0.001; η_p_^2^ = 0.063), and the CFC-14 Immediate scale (*F*_1, 254_ = 7.39; *p* =  < 0.01; η_p_^2^ = 0.028), with males outperforming females.

To ascertain whether there were associations between age, years of education, SOGS, GRCS, and CFC-14 scores, Pearson’s correlation coefficients were calculated. The results showed strong to moderate associations among the variables (see Table 2).

**Table 2:** Pearson correlation coefficients among age, years of education, SOGS total score, GRCS and CFC-14 subscales scores, and chasing frequency

	2	3	4	5	6	7	8	9	10
1. Age	.253**	− .127*	− .203**	− .107	− .198**	− .107	− .184**	.108	− .042
2. Education	–	− .168**	− .112	− .177**	− .282**	− .107	− .144*	− .177**	.049
3. SOGS		–	.559**	.531**	.593**	.566**	.594**	.154*	.045
4. Generalized Expectancies			–	.608**	.633**	.695**	.711**	.192**	.053
5. Illusion of Control				–	.741**	.683**	.701**	.311**	-.036
6. Predictive Control					–	.614**	.744**	.326**	.069
7. Inability to Stop gambling						–	.714**	.259**	-.057
8. Interpretative Bias							–	.253**	.094
9. CFC Immediate								–	.010
10. CFC Future									–

*p < .05; **p < .01

To establish whether, as hypothesized, the decision to quit or continuing gaming (i. e. the choice to chase) after the first phase and chasing frequency (i. e. the total number of trials played during the second phase) varied according to the experimental condition, data were submitted to chi-square test and univariate ANOVA. Chi-square test indicated that the choice to play further did not vary as a function of experimental condition (χ^2^(2, N = 255) = 0.576; ns), whereas the results of ANOVA showed a significant difference due to experimental condition in chasing frequency (*F*_2, 252_ = 3.15; *p* < 0.05; η_p_^2^ = 0.024). Bonferroni post-hoc test (*p* < 0.05) revealed that, compared to control group, participants in the Loss condition chased significantly more often. However, no difference in chasing frequency was observed between the Loss and the Win condition.

To assess the relative contribution of gender, age, education (step 1), experimental condition after dummy coding (step 2), cognitive distortions related to gambling (GRCS scores), temporal perspective (CFC-14 scores), and gambling severity (SOGS total score) (step 3) for the choice to chase, a hierarchical logistic regression analysis was conducted, using the two groups (chasers and nonchasers) as the criterion variable. For the regression, the Hosmer and Lemeshow’s test was not significant [χ^2^(8, N = 255) = 11.24; *p* = 0.19], indicating an adequate model fit. The results of the final regression model showed that scores on the GE and IB subscales of the GRCS and on the CFC-14 Immediate dimension were significant predictors of the choice to chase (see Table 3). Notably, gambling severity (SOGS total score) was not included in the final model.

**Table 3:** Results of the final logistic regression model

	*B*	*SE*	Wald	*df*	*p*	Odds ratio (95% CI)
CFC-14 I^a^	.072	.022	10.836	1	.001	1.075 (1.030-.1.122)
GRCS GE^b^	.896	.370	5.860	1	.015	2.449 (1.186–5.057)
GRCS IB^c^	.841	.302	7.767	1	.005	2.318 (1.283–4.186)

Dependent variable: Group (nonchasers/chasers)

Model: χ^2^ = 68.99; Nagelkerke’s *R*^2^ = .343. Overall percentage accuracy rate = 78.8%

^a^Consideration of Future Consequences scale: Immediate; ^b^Gambling Related Cognitions Scale: Generalized Expectancies; ^c^Gambling Related Cognitions Scale: Interpretative Bias

Finally, chi-square test was used to ascertain whether there was a relationship between the choice to chase and each motive for gambling. Although about a third of the participants reported they gamble mainly for winning money (34.5%) or for fun (25.1%), the results revealed that, relative to nonchasers, chasers continue to play significantly more to gain money [χ^2^(1, N = 255) = 10.24; *p* < 0.01; Cramér’s *V* = 0.20].

To identify the potential predictors of chasing frequency, gender, age, education (in years), experimental condition (after dummy coding), scores on SOGS, GRCS, and CFC-14 were input to a hierarchical regression analysis with chasing frequency as the dependent measure. The results (see Table 4) showed that, along with the Loss condition, GE, PC, and CFC-Immediate scores were significant predictors of chasing frequency (*R*^2^_adj_ = 0.32, *F*_5, 249_ = 24.4; *p* < 0.001). Again, SOGS total score was not retained in the final regression model.

**Table 4:** Summary of hierarchical linear regression analysis with Chasing total score as the dependent variable

Variable	B	*R*^2^	*ΔR*^2^	β	*t*	*p*	VIF
*Step 1*							
Education	−.030	.015	.015	−.123	−1.970	.050	1.000
*Step 2*							
Education	−.029	.036	.021	−.120	−1.935	.054	1.000
Loss condition	.279			.145	2.338	.020	1.000
*Step 3*							
Education	−.016	.269	.233	−.065	−.1196	.233	1.013
Loss condition	.290			.150	2.785	.006	1.001
GRCS GE^a^	.770			.486	8.954	.000	1.013
*Step 4*							
Education	−.002	.307	.038	−.009	−.171	.865	1.095
Loss condition	.262			.136	2.5743	.011	1.006
GRCS GE^a^	.517			.326	4.773	.000	1.688
GRCS PC^b^	.310			.262	3.693	.000	1.815
*Step 5*							
Education	.001	.329	.022	.005	.092	.927	1.105
Loss condition	.277			.143	2.747	.006	1.009
GRCS GE^a^	.521			.329	4.878	.000	1.689
GRCS PC^b^	.252			.213	2.957	.003	1.926
CFC-14 I^c^	.019			.156	2.831	.005	1.132

B: unstandardized coefficient; *ΔR*^2^: *R* square change; β: standardized regression

Coefficient; VIF: Variance Inflation Factor

^a^Gambling Related Cognitions Scale: Generalized Expectancies; ^b^Gambling Related Cognitions Scale: Predictive Control; ^c^Consideration of Future Consequences scale: Immediate

**Fig. 1:**
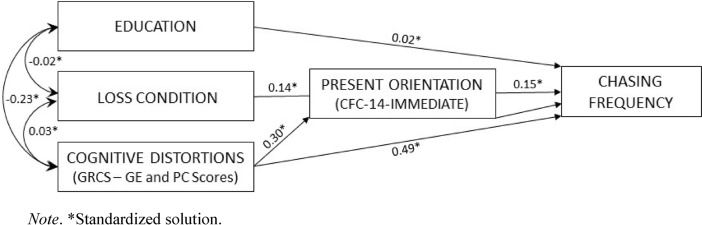
Path diagram for Model 2

Finally, to ascertain if cognitive distortions related to gambling were the mediator of the impact of present orientation on chasing propensity (assessed through chasing frequency) or if present orientation (high scores on the CFC-14 Immediate subscale) was on the path from gambling-related cognitions to chasing proneness, we compared two different models: the former (Model 1) assumed that high scores on the CFC-14 Immediate subscale can predict chasing not only directly, but also indirectly via high scores on the GE and PC subscales of the GRCS; the latter (Model 2) assumed that cognitions related to gambling can predict chasing not only directly, but also indirectly via present orientation. For both models a composite cognitive distortion score, obtained as the sum of the scores on the GE and PC subscales, was used. Model fit statistics (GFI and CFI estimates, RMSEA and SRMR values) for the two models are displayed in Table 5 and the path diagram is reported in Fig. 1. As Table 5 shows, relative to the first model, the second one fit better to the data.

**Table 5:** Path analysis fit indexes for alternative models

	S-B χ^2^	*df*	GFI	CFI	RMSEA [90% CI]	SRMR
MODEL 1	9.65	2	.99	.94	.123 [.053, .205]	.053
MODEL 2	3.93	2	.99	.99	.062 [.000, .152]	.030

S-B χ^2^ = Satorra-Bentler scaled χ^2^ statistic; GFI = Goodness of Fit Index; CFI = Comparative Fit Index; RMSEA = Root-Mean-Square Error of Approximation; 90% CI = 90% confidence interval for RMSEA; SRMR = Standardized Root-Mean-Square Residual

As Fig. 1 shows and covariances among independent variables indicate, education was negatively associated to GRCS scores (Z = 3.59; *p* < 0.05). No significant associations were found between educational level and the Loss condition and between Loss condition and GRCS scores, respectively. After the decomposition of the effects, Beta values indicated that the total effects of the GRCS, the CFC-14 Immediate scores on chasing frequency were all significant (all *ps* > 0.05). The indirect effect of GRCS and CFC-14 Immediate scores on chasing scores frequency were both significant as well (Z = 2.37; *p* < 0.05 and Z = 2.69; *p* < 0.05).

## Discussion

This study first empirically investigated the interplay between gambling severity, gambling-related cognitions, temporal perspective, and chasing behavior in a sample of adult habitual gamblers. The results of logistic regression analysis showed that the choice to stop or continue playing depended on cognitive biases and positive expectancies related to gambling and present orientation. The results of hierarchical linear regression analysis indicated that chasing proneness depended on experimental condition, high scores on both the GRCS subscales Gambling Expectancies (GE) and Predictive Control (PC), as well as on present orientation. Moreover, as the results of path analysis indicated, high scores on the GE and PC dimensions of the GRCS predict chasing frequency directly, as well as indirectly via high scores on the CFC-14 Immediate scale. More interestingly, in both regression analyses, gambling severity (SOGS total score) was not included in the final models. This finding further supports the idea that, *ceteris paribus*, chasers and nonchasers belong to two quite different categories of gamblers (Ciccarelli et al., 2019a; James et al., 2016; Kong et al., 2014; Nigro et al., 2018a, 2018b, 2019).

About the role of experimental condition, the results we obtained did not indicate significant differences in chasing frequency between chasing losses and chasing wins. This finding dovetail with the results reported by Dickerson (2003) and Lister et al. (2016) and suggests that playing in the attempt to recoup lost money motivates to chase. Indeed, a recent study on chasing motivations revealed that the choice to continue playing after a series of losses is mostly driven by the hope of recouping lost money, whereas persisting in gambling after a series of wins (Win condition) is largely driven by the hope of further wins (Nigro et al., 2019).

Regarding gambling-related cognitions, the results showed that gambling expectancies predict both the choice to chase and chasing frequency. In general, gambling expectancies involve the erroneous belief that gambling will make one feel better. In this sense gambling expectancies resemble *positive illusions* (Taylor & Brown, 1988). Indeed, as noted by Raylu and Oei (2004), people gamble, among others, to relieve negative emotional states, to reduce boredom, to try to bet the odds, to have fun or excitement. Individuals who agree with the GRCS statements, such as “*Gambling makes me happier*”, “*Gambling makes the future brighter*”, or “*Having a gamble helps reduce tension and stress*” feel that gambling serves as an escape strategy from everyday life, to the point that renouncing gambling is perceived as deprivation and threat to wellbeing. According to Ledgerwood et al. (2020), “gambling-related cognitive distortions appear to relate to baseline gambling severity in a linear fashion; as severity of gambling behavior rises, the intensity of gambling-related cognitive distortions increases” ( p. 670). Paraphrasing Ledgerwood et al. (2020), we found that as chasing behavior increases, the intensity of gambling-related cognitive biases grows, even apart from gambling severity.

The decision to play further was also predicted by high scores on the GRCS dimension Interpretative Biases. The erroneous perception of ability to interpret or control ambiguous events leads to believe that wins depend on personal skills (“*Relating my winnings to my skill and ability makes me continue gambling*”), and losses are related to bad luck (“*Relating my losses to bad luck and bad circumstances makes me continue gambling*”). In other words, and paradoxically, two opposites seem to coexist in gamblers: an internal as well as an external locus of control (Donati et al., 2015). Believing that wins depend on one's own skills and, at the same time, attributing losses to uncontrollable external forces (such as fate or bad luck) clearly bring out this paradox. Such self-serving bias (Goodie et al., 2019), inevitably encourage continued gambling despite previous losses.

The results of linear regression analysis indicated that chasing proneness was predicted not only by high scores on the GRCS GE dimension, but also by high scores on the Predictive Control scale that reflects cognitions related to the ability to predict gambling outcomes. It is not surprising this association between the illusion of predictive control over gambles and chasing. Indeed, assuming that “*Losses while gambling are bound to be followed by a series of wins*”, “*When I have a win once, I will definitely win again”* (hot-hand fallacy), “*I have some control over predicting my gambling wins*” (gambler’s fallacy) and are quintessential features of chasing behavior. Again, the results we obtained showed a strong association between the persistence in chasing and the illusory control over gambling outcomes, over and above gambling severity.

Consistent with previous research (see Ciccarelli et al., 2019a for a review) our results indicated that both the decision to chase and chasing frequency depend also on myopia for the future. In our study, relative to nonchasers, chasers showed to be more oriented to the present, rather than thinking about the future. The decision to bet again in the hope of recoup previous losses “leads gamblers to make apparently fruitful choices in the short-term that turn out to be of dubious value in the long-term. Indeed, most of the time, the attempt to recoup losses fails and results in the accumulation of further losses, triggering a vicious circle that can lead to a loss of control of gambling activity” (Ciccarelli et al., 2019a, p. 264).

More interestingly, the results of path analysis suggest that gambling-related cognitions affect chasing frequency not only directly, but also indirectly via high scores on the CFC-14 Immediate scale. It may be that some irrational ways of thinking contribute to restrict temporal perspective. Paradoxically, believing that gambling outcomes can be predicted based on previous patterns fosters the illusion that the future is somewhat controllable. Perceiving that gambling will make one feel better and assuming that game outcomes are to certain extent under control could fuel present orientation.

Overall, these results suggest that the decrease of chasing behavior could potentially be facilitated by psychotherapeutic interventions focused both on reducing gambling-related cognitive distortions and training people to think about the future. As stated by Ledgerwood et al. (2020), cognitive distortions are important “for both understanding of this mechanism of gambling disorder, and for identifying distortions as potential targets of gambling treatment” (p. 681). In our opinion, cognitive distortions could be equally useful to shed light on the mechanism underlying chasing propensity. Since our results indicated that gambling severity did not play a central role in chasing behavior, although the opposite is true, a promising avenue in gambling disorder treatment could be tailoring the intervention on different subtypes of gamblers, such as nonchasers and chasers.

## Limitations

Although several strengths characterized this study, including a large sample and the use of a behavioral task to assess chasing, some limitations need to be acknowledged. First, the participants were recruited using convenient sampling of Italian habitual players. Second, the current data are mainly based on self-report measures, which may limit the generalizability of the results. Furthermore, gambling severity was assessed through a measure that has been criticized for excessive false positives (Goodie et al., 2013). However, it is worth noting that the SOGS demonstrated satisfactory reliability and validity (Stinchfield, 2002). Despite these limitations, to the authors' knowledge, the present study is the first to investigate the interplay between chasing behavior, gambling-related cognitions, and time perspective among habitual players.

Future research should be addressed to analyze more deeply the interplay between shortened time horizon and gambling-related cognitive distortions in chasing behavior and (perhaps more importantly) focus on testing ad hoc treatments for disordered gamblers with or without chasing propensity.
